# The six most essential questions in psychiatric diagnosis: a pluralogue part 3: issues of utility and alternative approaches in psychiatric diagnosis

**DOI:** 10.1186/1747-5341-7-9

**Published:** 2012-05-23

**Authors:** James Phillips, Allen Frances, Michael A Cerullo, John Chardavoyne, Hannah S Decker, Michael B First, Nassir Ghaemi, Gary Greenberg, Andrew C Hinderliter, Warren A Kinghorn, Steven G LoBello, Elliott B Martin, Aaron L Mishara, Joel Paris, Joseph M Pierre, Ronald W Pies, Harold A Pincus, Douglas Porter, Claire Pouncey, Michael A Schwartz, Thomas Szasz, Jerome C Wakefield, G Scott Waterman, Owen Whooley, Peter Zachar

**Affiliations:** 1 Department of Psychiatry, Yale School of Medicine, 300 George St, Suite 901, New Haven, CT, 06511, USA; 2 Department of Psychiatry and Behavioral Sciences, Duke University Medical Center, 508 Fulton St, Durham, NC, 27710, USA; 3 Department of Psychiatry and Behavioral Neuroscience, University of Cincinnati College of Medicine, 260 Stetson Street, Suite 3200, Cincinnati, OH, 45219, USA; 4 Department of History, University of Houston, 524 Agnes Arnold, Houston, 77204, USA; 5 Department of Psychiatry, Columbia University College of Physicians and Surgeons, Division of Clinical Phenomenology, New York State Psychiatric Institute, 1051 Riverside Drive, New York, NY, 10032, USA; 6 Department of Psychiatry, Tufts Medical Center, 800 Washington Street, Boston, MA, 02111, USA; 7 Human Relations Counseling Service, 400 Bayonet Street Suite 202, New London, CT, 06320, USA; 8 Department of Linguistics, University of Illinois, Urbana-Champaign, 4080 Foreign Languages Building, 707S Mathews Ave, Urbana, IL, 61801, USA; 9 Duke Divinity School, Box 90968, Durham, NC, 27708, USA; 10 Department of Psychology, Auburn University Montgomery, 7061 Senators Drive, Montgomery, AL, 36117, USA; 11 Department of Clinical Psychology, The Chicago School of Professional Psychology, 325 North Wells Street, Chicago, IL, 60654, USA; 12 Institute of Community and Family Psychiatry, SMBD-Jewish General Hospital, Department of Psychiatry, McGill University, 4333 cote Ste. Catherine, Montreal, H3T1E4, QC, Canada; 13 Department of Psychiatry and Biobehavioral Sciences, David Geffen School of Medicine at UCLA, 760 Westwood Plaza, Los Angeles, CA, 90095, USA; 14 VA West Los Angeles Healthcare Center, 11301 Wilshire Blvd, Los Angeles, CA, 90073, USA; 15 Department of Psychiatry, SUNY Upstate Medical University, 750 East Adams St, #343CWB, Syracuse, NY, 13210, USA; 16 Irving Institute for Clinical and Translational Research, Columbia University Medical Center, 630 West 168th Street, New York, NY, 10032, USA; 17 New York Presbyterian Hospital, 1051 Riverside Drive, Unit 09, New York, NY, 10032, USA; 18 Rand Corporation, 1776 Main St Santa Monica, California, 90401, USA; 19 Central City Behavioral Health Center, 2221 Philip Street, New Orleans, LA, 70113, USA; 20 Center for Bioethics, University of Pennsylvania, 3401 Market Street, Suite 320, Philadelphia, PA, 19104, USA; 21 Department of Psychiatry, Texas A & M College of Medicine, 4110 Guadalupe Street, Austin, Texas, 78751, USA; 22 Silver School of Social Work, New York University, 1 Washington Square North, New York, NY, 10003, USA; 23 Department of Psychiatry, NYU Langone Medical Center, 550 First Ave, New York, NY, 10016, USA; 24 Department of Psychiatry, University of Vermont College of Medicine, 89 Beaumont Avenue, Given Courtyard N104, Burlington, Vermont, 05405, USA; 25 Institute for Health, Health Care Policy, and Aging Research, Rutgers, The State University of New Jersey, 112 Paterson St, New Brunswick, NJ, 08901, USA

## Abstract

In face of the multiple controversies surrounding the DSM process in general and the development of DSM-5 in particular, we have organized a discussion around what we consider six essential questions in further work on the DSM. The six questions involve: 1) the nature of a mental disorder; 2) the definition of mental disorder; 3) the issue of whether, in the current state of psychiatric science, DSM-5 should assume a cautious, conservative posture or an assertive, transformative posture; 4) the role of pragmatic considerations in the construction of DSM-5; 5) the issue of utility of the DSM – whether DSM-III and IV have been designed more for clinicians or researchers, and how this conflict should be dealt with in the new manual; and 6) the possibility and advisability, given all the problems with DSM-III and IV, of designing a different diagnostic system. Part 1 of this article took up the first two questions. Part 2 took up the second two questions. Part 3 now deals with Questions 5 & 6. Question 5 confronts the issue of utility, whether the manual design of DSM-III and IV favors clinicians or researchers, and what that means for DSM-5. Our final question, Question 6, takes up a concluding issue, whether the acknowledged problems with the earlier DSMs warrants a significant overhaul of DSM-5 and future manuals. As in Parts 1 & 2 of this article, the general introduction, as well as the introductions and conclusions for the specific questions, are written by James Phillips, and the responses to commentaries are written by Allen Frances.

## General introduction

For the full text of the General Introduction to the entire article, the reader is referred to Part 1 [1]. The General Introduction reviewed the history of the article, which originated in a controversy initiated by Robert Spitzer and Allen Frances, Chairmen respectively of the DSM-III and DSM-IV Task Forces, over the ongoing work of the DSM-5 Task Force and Work Groups. In a series of articles and blog postings in *Psychiatric Times,* Frances (at times with Spitzer) carried out a sustained critique of the DSM-5 work in which he focused both on issues of transparency and issues of process and content [2-15].

In the course of this debate over DSM-5 I proposed to Allen in early 2010 that we use the pages of the Bulletin of the Association for the Advancement of Philosophy and Psychiatry (of which I am Editor) to expand and bring more voices into the discussion. This led to two issues of the Bulletin in 2010 devoted to conceptual issues in DSM-5 [16,17]. (Vol 17, No 1 of the AAPP Bulletin will be referred to as Bulletin 1, and Vol 17, No 2 will be referred to as Bulletin 2. Both are available at http://alien.dowling.edu/~cperring/aapp/bulletin.htm.) Interest in this topic is reflected in the fact that the second Bulletin issue, with commentaries on Frances’ extended response in the first issue, and his responses to the commentaries, reached over 70,000 words.

Also in 2010, as Frances continued his critique through blog postings in *Psychiatric Times*, John Sadler and I began a series of regular, DSM-5 conceptual issues blogs in the same journal [18-31].

With the success of the Bulletin symposium, we approached the editor of PEHM, James Giordano, about using the pages of PEHM to continue the DSM-5 discussion under a different format, and with the goal of reaching a broader audience. The new format would be a series of “essential questions” for DSM-5, commentaries by a series of individuals (some of them commentators from the Bulletin issues, others making a first appearance in this article), and responses to the commentaries by Frances. Such is the origin of this article. (The general introduction, individual introductions, and conclusion are written by this author (JP), the responses by Allen Frances.

For this exercise we have distilled the wide-ranging discussions from the Bulletin issues into six questions: 1) the nature of a mental disorder; 2) the definition of mental disorder; 3) the issue of whether, in the current state of psychiatric science, DSM-5 should assume a cautious, conservative posture or an assertive, transformative posture; 4) the role of pragmatic considerations in the construction of DSM-5; 5) the issue of utility of the DSM – whether DSM-III and IV have been designed more for clinicians or researchers, and how this conflict should be dealt with in the new manual; and 6) the possibility and advisability, given all the problems with DSM-III and IV, of designing a different diagnostic system. Part 1 [1] of this article covered the first two questions, and Part 2 [32] the second two questions. This text, Part 3, covers the fifth and six questions. The final Part 4 of the article will contain the general conclusion.

### Question #5: How compatible are all the purposes of DSM?

*Is there a conflict over utility in the DSMs? The authors of DSM-III, DSM-IV, and DSM-5 intend the manuals to be useful for both clinicians and researchers. Is there a conflict between what is useful for clinicians and what is useful for researchers? Which group is served better by DSM-III and DSM-IV, and by the prospective changes in DSM-5?*

## Introduction

Any discussion of the utility question in DSM-5 should begin with the way in which the previous manuals dealt with this question. The first paragraph of the Introduction to *DSM-IV* contains the following statements: “The utility and credibility of *DSM-IV* require that it focus on its clinical, research, and educational purposes and be supported by an extensive empirical foundation. Our highest priority has been to provide a helpful guide to clinical practice. We hoped to make *DSM-IV* practical and useful for clinicians by striving for brevity of criteria sets, clarity of language, and explicit statements of the constructs embodied in the diagnostic criteria. An additional goal was to facilitate research and improve communication among clinicians and researchers” ([33], p. xv).

The primary achievement of DSM-III and DSM-IV was the establishment of reliability through the use of diagnostic criteria. The criteria worked to assure consistency of diagnosis across settings. Psychiatrists using the same diagnosis had the comfort of knowing that they were talking about the same patients - or that two psychiatrists evaluating the same patient would make the same diagnosis. As sensible as this rationale is, it leaves questions open - who in fact actually uses the diagnostic criteria, and for whom are they really important?

In discussing these questions, we should not forget that the DSM manuals also provide thorough descriptions of the respective disorders, what we may call descriptive, phenomenological, or prototypal accounts. Among the various user groups - clinicians, students, and researchers, to name the three cited in the above quotation from the DSM-IV Introduction - who uses what? The available research [34] supports one’s anecdotal impression that experienced clinicians use the descriptive prototypes, calling on the criteria for the occasional infrequently used diagnosis, and that students and researchers are the main users of the criteria. This is hardly surprising. Clinicians are focused on treating their patients, not checking criteria, and are usually working with a mental grasp of the prototype; students are focused on learning the diagnoses; and researchers are required to be careful that their research subjects meet the criteria.

It is difficult to avoid the conclusion that the diagnostic criteria are mainly useful for researchers, who are obligated to insure a uniform research population. In this regard, it is an ironic side effect of the diagnostic criteria that they may impede research by confining research efforts to criteria-determined questions [35].

The introduction of dimensional measures into DSM-5 adds a further wrinkle to this discussion. Regier and colleagues have written: “The single most important precondition for moving forward to improve the clinical and scientific utility of DSM-5 will be the incorporation of simple dimensional measures for assessing syndromes within broad diagnostic categories and supraordinate dimensions that cross current diagnostic boundaries. Thus, we have decided that one, if not the major, difference between DSM-IV and DSM-5 will be the more prominent use of dimensional measures in DSM-5” [36] (see also [37]). These authors assure us once again that these dimensions will provide clinical as well as scientific utility. The proposed dimensional measures raise two questions: should they even be in the manual, and who will use them. The questions raised above about who will use them are apposite here. Given their undemonstrated scientific status, Frances challenged their introduction into DSM-5 in Bulletin 2, and Paris and Whooley challenge it below. First and colleagues have argued that any change in the existing manual should use clinical utility as a criterion of change [38], and First has expressed concern over the dubious clinical utility of the proposed measures [39].

### Commentary: Useful for whom? clinicians, researchers, and DSM’s many-sided nature

Owen Whooley, Ph.D.

Institute for Health, Health Care Policy, and Aging Research

Rutgers University

The DSM serves many interests and performs many tasks. It provides a common language of mental disorders for clinicians, standardized categories for researchers, diagnostic codes for insurance companies, and valid diagnoses of mental illness for legal and juridical purposes. Task Forces of DSMs, past and present, have treated these tasks as complimentary, but their diversity belies the inherent tensions in the complex negotiation of these interests.

There is no tension more challenging than that between psychiatric clinicians and researchers. The introductions to each edition of the DSM state that the “highest priority” of the manual is “to provide a helpful guide to clinical practice,*”* ([33], p. xxiii). Yet, the manual is produced by psychiatric researchers, who are attuned to different goals (e.g. reliability, standardization, robust research designs, and statistical analyses) than the average clinician. Indeed clinicians are often portrayed as obstacles to these goals as evident in the discussion around DSM-5. Framed as an issue of “clinical utility”, this discussion focuses on the mundane, practical limitations to reforming the clinical practice - the extra paperwork, the pressing lack of time, the need for computer access, and the unmanageability of 38-point personality scales. Clinicians, according to this view, need to be accommodated by the Task Force, which is charged with the impossible task of achieving a paradigm shift with as little disruption as possible.

While these technical problems are real, the Task Force reads them incorrectly. Misconstrued as a technical problem, the tension between researchers and clinicians in psychiatry has deep roots that no clever construction of new dimensional scales can solve. Rather than the specific exigencies of clinical practice, the salient issue is epistemological in nature. Quite simply, the divergent roles that clinicians and researchers serve in the profession lead to different orientations toward knowledge and competing models of what constitutes useful psychiatric knowledge. It is these differences that underwrite the debates over clinical utility.

The epistemological tensions between researchers and clinicians reflect a classic distinction, noted by Aristotle, between *episteme* and *phronesis*[40]. *Episteme* is what we now understand as scientific reasoning; its goal is to illuminate universal and general rules, to uncover timeless Truth. This is how psychiatric researchers understand the purpose of psychiatric knowledge. Since DSM-III, each edition of the DSM – including and especially DSM-5 - has aimed for the standardization of psychiatric knowledge. The continual push for more reliability in diagnosis is animated by a concurrent desire to reign in idiosyncratic, subjective interpretations of clinicians, so that researchers can accumulate objective data and achieve generalizable knowledge on mental disorders. Or in the words of DSM-III ([41], p. 7), researchers promote a standardized classification system “with the lowest order of inference necessary”.

Clinicians, on the other, understand relevant psychiatric knowledge differently, adopting a more practical posture toward knowing. Practical wisdom, or what Aristotle calls *phronesis,* addresses particular cases and specific quandaries, employing, not maxims or rules, but a network of considerations to be tested by trial and error. It operates in the realm of the concrete, the temporal and the presumptive. The goal is not universal knowledge for its own sake, but practical *intervention* through case-based reasoning. In the *phronesis* of psychiatric practice, clinicians are not interested in identifying a universal truth but a particular one – what will work for a specific patient.

*Phronesis* and *episteme* are not inherently opposed. In other branches of medicine this divide is bridged by concrete diagnostic tests and treatment protocols, backed by research, which then inform – but do not dictate – clinical practice. But, as of now, psychiatry lacks these bridges, and simply reforming a nosological manual will not provide one, as it skewed toward standardization at the expense of clinical intuition.

The recommendation of the DSM-5 Task Force to add dimensional measurements to the current focus on diagnostic categories is likely to widen the divide between clinicians and researchers. In pivoting toward a more dimensional model of mental illness, the Task Force has embraced a project of quantification by introducing numerical scales. These scales value information of a specific kind, namely statistical, quantitative data. They aim to transform the complex suffering of individual patients into numerical values. In quantification, researchers shoot for more standardization via quantification in the interest of obtaining information on aggregate populations of patient.

This is at odds with the practical motives of clinicians. Clinicians are oriented toward individual patients. Statistical aggregates are of little use or relevance to them. The patient can never be just a number. For clinicians, the trade-off for this marginally useful statistical knowledge is not only more bureaucratic headaches; it is an attack on clinical discretion– an attempt to standardize the patient/psychiatrist intervention that devalues the nuance and particularistic thinking required for treating patients. Dimensionalization carries with it great implications for identity of clinicians as knowers. It threatens to alter the role of psychiatrists from interpreters to measurers, as the dimensional revisions currently being field tested (i.e. cross-cutting dimensional scales and severity scales for each diagnosis) will, in effect, bookend the clinical interaction with the production of numerical data. The goal of reigning in discretion is evident in a major justification for dimensionalization - to *reduce* the need for clinical judgment in diagnosis so as to assist non-psychiatrists ([34], p. 21). Such diagnosis by numbers threatens the hermeneutic tradition of the psychiatric practice, by promoting *episteme* at the expense of *phronesis*.

The effort to standardize clinical practice through the DSM fosters ambivalence (at best) and/or resentment (at worst) in clinicians. It is perhaps unsurprising then that many clinicians resist DSM reforms. In fact, this epistemological divide probably underlies clinicians’ long-standing refusal to follow DSM protocols lock-step. DSM reformers lament clinicians’ rejection of the GAF, their refusal to adopt the multiaxial system, and the prevalent use of vague, “garbage can” diagnoses like NOS category. Often viewed as technical deficiencies with the DSM itself (i.e. Regier et al. [36]), these practices might be better conceived of as ways in which clinicians resist the imposition of the DSM. These “workarounds” are assertions of *phronesis*, an attempt to carve out a space of autonomy from the DSM in practice [42].

Thus, on the eve of DSM-5, clinicians find themselves in a complicated relationship with the DSM that dimensionality is poised to worsen. On one hand, as members of a profession, clinicians gain credibility from their association with science and research. The DSM has played an essential role in linking psychiatry to science for the last thirty years. On the other hand, the more the needs of researchers dictate the DSM and impinge on clinical practice, the more clinical intuition is devalued in those contexts. Scales may obtain more advanced statistical analyses and knowledge but devalue clinical nuance in the process.

Researchers and clinicians do not see eye-to-eye on dimensionality because their gazes are directed at different targets, aggregate collectives and individual patients, respectively. Rather than a bridge, dimensionality erects a barrier between the *episteme* of researchers and the *phronesis* of clinicians. The existence of this epistemological divide raises a question: can the DSM center hold? Instead of hitching together to epistemologically divergent interests, should psychiatry decouple the efforts of researchers and clinicians so that a bridge may arise, not by fiat, but more organically?

## Commentary

Joseph Pierre, M.D.

UCLA Department of Psychiatry

Note that part of the reason we don’t understand enough about psychiatric pathophysiology is that etiologic research typically begins with a patient population defined by a DSM diagnosis. Indeed, DSM-III was borne out of specific efforts to improve reliability for research studies. But post-DSM-III etiologic research has yielded mostly inconsistent or heterogeneous findings, without discovering any kind of “lesion” to account for psychiatric disorders. That research hasn’t been a waste – it has provided important clues about pathophysiology, along with bigger-picture evidence that DSM diagnoses are constellations of symptoms for which there are probably myriad etiologies.

While this conclusion has been cited as a justification to create a new DSM-5 (i.e. if we redefine the diagnostic criteria, then we can do a better job of “finding a lesion”), that kind of thinking is wrong-headed. Diagnostic revision should follow, not precede, etiologic discoveries – in other words, a new DSM-5 needs etiologic discoveries, but etiologic discoveries do not need a new DSM. Indeed, novel directions in etiologic research have been ongoing for some time, and are embodied in the NIMH’s Research Domain Criteria Project that recognizes the complexity of psychiatric illnesses as dysregulations of neural networks and focuses its pathophysiologic spotlight, not so much on DSM disorders, but on endophenotypes and dimensions of symptoms both within and across disorders [43]. When and if those research efforts come to fruition, it will be time to welcome a radical new revision of the DSM.

In the meantime, etiologic research in particular must operate outside the bounds of DSM-IV, and will not greatly benefit from the proposed revisions for the DSM-5. In this way, there is a conflict over utility between clinicians and researchers, and one manual cannot satisfy both goals [44]. But that is not to say that all psychiatric research should disengage from the DSM – on the contrary, clinical research (i.e. therapeutic trials) based on DSM disorders is a continued necessity to guide clinical practice. Such complexities arising from the different intended applications of the DSM indicate how important it is to consider “contextual utility” [45] when deciding whether or how DSM should evolve.

### Commentary: The DSMs: A wedge between clinician and clinical researcher?

Aaron L. Mishara, Ph.D.

The Chicago School of Professional Psychology

Michael A. Schwartz, M.D.

Texas A & M University Department of Psychiatry

In response to question # 5, we propose that beginning with DSM-III there is a decided conflict between what is useful for clinicians and researchers. However, this need not be the case. This conflict has more to do with the built-in agenda of the DSMs following DSM-III than with the nature of either diagnosis or research. We propose some fruitful ways for the two to work together.

### Learning from history: DSM-III’s research agenda

DSM-III’s [46] revolutionary neo-Kraeplinians were dedicated to setting up a research program rather than accurately reflecting clinical realities. Embracing Carl Hempel’s [47] logical empiricist agenda, they approached mental disorders in terms of operational definitions for the purpose of enhancing reliability in diagnosis [48]. Mayes and Horwitz [49] write: "Spitzer selected a group of psychiatrists and consultant psychologists who were committed primarily to medically oriented, diagnostic research and not to clinical practice" (p. 259). That is, **there was and remains a divide between DSM-III and later DSM's prescriptive diagnostic practices for researchers and what clinicians actually do in practice.** Far from bridging clinical practice and clinical research, DSM- III inserted a wedge between clinician and clinical researcher. Millon [50] writes: “Operational definitions are too restrictive. They preclude extensions to new situations that are even slightly different from the original defining condition” (p. 249). The original criteria used as the initial basis for the specified diagnostic criteria for the major diagnostic categories of DSM-III were regarded exclusively as “research diagnostic criteria” (RDCs), e.g., Williams and Spitzer [51]. In their zeal for reliable diagnosis, DSM-III advocates overlooked that the Hempelian approach they adopted was only one approach that neglected more phenomenologic approaches [52].

### Diagnosis is not a checklist but an interactive, embodied social cognitive process

Schwartz and Wiggins [53] proposed that clinicians in their practice use a different approach than that outlined by the neo-Kraeplinian embrace of Hempelian nomological science: the clinician's experience is already pervaded by typifications which help to structure the clinicians’ diagnosis meaningfully. The founder of philosophic phenomenology, Husserl, had indicated that perceptual meaning is itself based on such a typification process. We never perceive the individual thing or person but always in terms of the type that implicitly subsumes it. We perceive the not yet known in terms of the known, i.e., in terms of the general type that is activated in the particular perception. With each view, there is built a reference to the next anticipated view based on past experience of this and similar objects. The references between aspects are anticipatory constraints, which are nevertheless open to revision or cancellation in their structure so that each aspect prefigures its successor in seamless transition as belonging to the same perceptual object.

As any other expertise, diagnostic decision making is informed by largely unconscious processes. There is a “gut feeling” which rapidly guides the expert to the most fitting response in completely new contexts or “situations” (cf. Millon’s comment above)? The philosopher Gadamer [54] calls this process “hermeneutics,” the “art” of “interpretative application,” *how* the rule is somehow optimally applied to the particular case. The well-known neurologist, Damasio finds this process to be governed by what he calls “somatic markers.” Here, bodily or gut feelings based on past experience subtly “bias” current decision making often in an unnoticed manner (for hermeneutics-somatic marker relationship, see Mishara [55]).

Bransford and colleagues [56] note that very often the experts themselves are unable to provide an account of the decision processes leading to expert judgment in the particular case. They cannot articulate the “tacit knowledge” that guides their practice. Developing this sort of expertise takes years of training, a repeated learning by doing in each case to the individual situation, i.e., a learning by examples, whereby the learning after a while becomes automatic. We see the same sort of learning underlying diagnostic practice [57].

### Paradigm shift: Phenomenological-clinical neuroscience

The phenomenologic approach prepares the way for a paradigm shift from the biomedical model of DSM-III, and subsequent DSMs, to a more “person centered medicine.” Citing the Spanish existential-philosopher Ortega y Gasset, “I am I and my circumstance,” Mezzich and colleagues write, “Respect for the patient’s autonomy, values, and dignity represents a fundamental recognition of his or her personhood, and an ethical imperative. Slowly these concepts are finding their way into evidence.” ([58], pp. 304–305)

By checking off symptoms, i.e., by focusing solely on whether the patient’s responses fulfill diagnostic criteria, we stop asking the patient what she or he experiences. We take interest only in the client’s responses to the extent that they fulfill our predefined operationalized diagnostic criteria. These operationalized responses exclude further exploration of the patient’s experience. By relying solely on the DSM, researchers and clinicians actually pre-empt the further research of how the patient’s subjective experience can be mapped onto underlying neural processes and therefore, how it can be treated. We believe that the proposed paradigm shift to a more phenomenological-clinical neuroscience will provide a more holistic, narrative, strength based (empowering), contextual, culturally sensitive approach and eventually, a new understanding of mental disorders.

### Toolbox or Pandora’s box: The elusiveness of human subjectivity

Recently, we critiqued the metaphoric toolbox: “the clinical researcher, Mary Phillips proposes a ‘psychiatric toolbox’ (i.e., neuropsychological tests, neuroimaging, genotyping) to develop disorder ‘biomarkers’ that are persistent, rather than state-dependent. This would obviate the phenomenological research of the patient’s subjective experience of the disorder. The danger will be, however, that we will define disorders in terms of what technologies we have available.” ([59], p. 61)

We advise similar caution when using this metaphor in the application of phenomenology to clinical neuroscience. There are numerous methodological and conceptual problems in claiming that a pre-reflective awareness of self (as “immediately” given in self-affection) provides a “toolbox” for clinical neuroscience [60]. In this regard we agree with Schwabe and Blanke [61] that toolbox constructs, e.g., putative prereflective self-awareness, are unable to account for disruption of self-experience in such phenomenologically complex neuropsychiatric symptoms as autoscopy. Autoscopy is “a loosely related complex of experiences in which one sees (or experiences) a ‘double’ as external to one’s current vantage point” [62]. Here there are “at least two simultaneous or rapidly alternating embodied perspectives” (p. 75), which cannot be accounted for by the putative “phenomenological” toolbox. This argument will be spelled out in more detail in a subsequent publication.

Rather, phenomenological clinical neuroscience must take a different direction which we [59] outlined in our previous responses to Allen Frances.

The two phenomenological psychiatrists, Klaus Conrad [63] (see Mishara [64]) and Henri Ey, employed the nineteenth century neurologist, Hughlings Jackson’s approach to classification in terms of describing and formalizing the subjective experience of the patient as a field of consciousness which is disrupted in its organizing activity precisely in response to the degree of severity of the underlying neurobiologic disturbance. … [They] anticipated the kind of modeling of mental disorders later done by neural networks, animal models, and drug challenge studies with healthy individuals in the following sense. If we are able to describe and verbally capture everyday ‘healthy’ consciousness in terms of its ‘field’ organization, then we are able to model disorders by seeing how this “field of consciousness” is specifically disrupted in a particular mental disorder (as we currently classify it) by disabling this or that component of consciousness. To elaborate on this analogy: Neural network models, for example, simulate mental disorders by “damaging” this or that part of the network. Similarly, animal models lesion a crucial part of circuitry and drug models alter neurotransmitter signaling. In each case, the mental disorder is ‘modeled’ by systematically removing or altering some aspect of healthy functioning thought to be implicated in the disorder. Similarly, these phenomenological psychiatrists begin with healthy waking consciousness and by ‘damaging’ or ‘removing’ healthy components of this consciousness (in as it were introspective, phenomenologic thought experiments, what Husserl called ‘imaginative variation’), attempt to produce the subjective experience of symptoms until they arrive at a plausible model. In this way, … both Conrad and Ey apply a Jacksonian hierarchical approach to nervous functioning in the organization of the patient’s ‘field of consciousness. ([59], p. 65)

It was Hughlings Jackson who proposed a two-tiered system for diagnosis – with one tier reserved for clinical practice and a second for research: “There are two kinds of classification of diseases: one scientific, generally called theoretical, for the advancement of knowledge; one empirical or clinical, for practice” ([65], p. 33). We propose that by using the patient’s subjective experience of ‘symptoms’ as a standard, there should be ongoing studies of bidirectional feedback between clinical practice and the diagnostic classifications operationalized by researchers to further refine these classifications([59], p. 62).

In conclusion, “phenomenology is not the antithesis to operationalism but precisely the step required to translate the patient’s subjective experience of symptoms, etc., into workable operationalizable hypotheses which can be quantifiably measured using the experimental methods of clinical neuroscience (see Mishara, [55], p. 64).

### Allen Frances responds: Serving many masters

Psychiatric classifications are like maps – and like maps there is no single best way of charting the territory. Depending on our purpose, we may prefer to use a geological map, or a political map, or a topographical map, or an economic map, or a climate map-or some combination. Similarly, it might have made sense to have different and complementary DSMs – one for clinicians, another for researchers, and others for education, for forensics, for billing, for gathering statistics, and so on. The DSM we have is a common denominator, with both the strengths and the weaknesses that derive from attempting to serve so many different masters.

Let's do the weaknesses first. DSM-IV is far too detailed for clinicians; not nearly detailed enough for researchers; too boring for students; too imprecise for use in the courtroom; too long for insurance coders. It easily could have been fashioned into five different classificatory maps, each addressed to a different function and a different set of users.

But much would have been lost in the translation. However imperfect the fit to all of its tasks, DSM-IV has the great strength of bringing one unifying language to all of them. Its great value in bridging the clinical research interface would be lost if clinicians and researchers were reading off different pages. Students might as well learn the system they will someday use even if the stilted prose is not much fun to read. DSM would have less clinical credibility were it written only for legal or insurance purposes.

DSM-IV does none of its jobs perfectly and its awkward fit certainly creates a variety of problems. Some clinicians refuse to learn DSM and stick to their own personal prototypes of disorders. Many epidemiological researchers ignore the requirement for clinical significance before making a psychiatric diagnosis and therefore report ridiculously high rates of mental illness in the general population. Some students take the DSM descriptions too literally and lose the patient as they evaluate the criteria. Lawyers often find loopholes because the language of DSM is frustratingly below legal requirements for precision. And so on.

But the unifying and synthesizing whole of DSM-IV is still worth much more than would be the accumulated sum of its individual parts. However imperfect, the DSM's special value is as a common denominator that avoids a Babel and is good enough (if admittedly not best) at each of its jobs.

**Response to Dr Whooley:** This is an erudite discussion of an important issue. I agree completely with his critique of the DSM-5 proposals for impossibly complex dimensional evaluations. These were concocted by research types who have no understanding of the needs of clinical practice. But however ridiculous the proposals, the DSM center will hold because clinicians will simply ignore what they can't possibly use. It is unfortunate that the DSM-5 dimensions were not done in a simple, clinically friendly way, but they will not do much harm beyond giving dimensions an unnecessarily bad name.

**Response to Dr Pierre**: I love this quote,"a new DSM needs etiologic discoveries, but etiologic discoveries don't need a new DSM." Etiological discoveries will follow their own pace uninfluenced by DSM.

**Response to Drs Mishara and Schwarz**: We agree that DSM-III represented a takeover by the research criterion approach of what had previously been a simple, intuitively applied clinical manual. We disagree on the impact. I think this was mostly to the good; DSM-III focused on the problems created by the existing manual. DSM-II was an essentially worthless document that represented a dead end for psychiatry. DSM-III was undoubtedly flawed in many ways, but it was also a salvation for psychiatry and a great help for its patients.

Spitzer's innovation was to apply his experiences creating the Research Diagnostic Criteria to the essentially clinical task of creating DSM-III. Only someone with Spitzer's vision and stubborn determination could have moved the field so far toward a criterion based system. DSM-III was absolutely essential in keeping psychiatric diagnosis relevant, in providing the possibility of reliable diagnosis, in furthering research, and in bridging the clinical/research interface.

The phenomenological approach advocated by Drs Mishara and Schwarz may provide a richer and more nuanced view of the individual, but one that is inherently inferential and unreliable. This is a useful listening style for the clinician trying to understand the patient's inner experience, but it is not a reliable guide to diagnostic decision making. "Gut feelings" are invaluable in therapists, often misleading for diagnosticians.

## Question #6: Is DSM the only way to do diagnosis?

*Given the problems in DSM-III, DSM-IV, and (likely) in DSM-5, would you argue for an alternative, more rational diagnostic system than the DSM? Could you describe it? Would your alternative system simply replace the DSM or restructure it in a major way?*

### Introduction

Should the activist stance toward change proposed by some commentators in question 3 be carried to another level – that of replacing the manual with an alternative, more rational one? Or, if not replacing the manual, of restructuring it substantially?

We can sort the possible responses to this question into three groups: alternative systems already in development, alternative nosological systems developed by the commentators themselves, and major restructuring proposals for the DSM. For a representative of the first group we have First’s presentation of the NIMH Research Domain Criteria Project (RDoC). In the second group belongs Pies’ proposal (along with those of Hayes, Mender, Mishara and Schwartz, Peled, and Pies in Bulletin 2). And to represent the third group we have Paris’ discussion of the thrust toward dimensional measures in DSM-5 (also covered by Whooley in the preceding question).

## Commentary: The national institute of mental health research domain criteria (RDoC) project: Moving towards an etiologically-informed diagnostic classification in psychiatry

Michael B. First, M.D.

Columbia University Department of Psychiatry

The diagnostic categories in the DSM and the ICD are defined in terms of syndromes, i.e., symptoms that cluster together and co-vary over time. When these were first introduced into DSM-III in 1980, it was widely assumed that although the identified psychiatric syndromes consisted entirely of descriptive symptoms, their underlying neurobiological mechanisms and pathophysiology would eventually be elucidated and that one day psychiatric disorders would be defined using objective laboratory findings, as is done in most of the rest of medicine. Unfortunately, however, after 30 years of intensive efforts by the research community, not a single biological marker or gene has been discovered that is useful in making a psychiatric diagnosis [66]. Although this frustrating lack of progress stems mostly from the fact that the problem of trying to understand the underlying etiology and pathophysiology of mental disorders has turned out to be much more complex than originally anticipated, it is likely that the categorical descriptive DSM system itself is at least partly to blame. Scientists attempting to discover the neurobiological or genetic underpinnings of psychiatric illnesses have all too often treated the man-made psychiatric constructs in the DSM as if they were “natural kinds,” looking for the gene for schizophrenia or the neurocircuitry underlying major depression as if they were real disease entities [67-69]. Perhaps whatever specificity there is between biological findings and behavioral correlates is being obscured by employing the DSM categories as if they were phenotypes, rather than focusing on more fundamental behavioral elements which cut across the various extant DSM categories.

The NIMH-sponsored Research Domain Criteria (RDoC) project is intended to establish “a framework for creating research classifications that reflect functional dimensions stemming from translational research on genes, circuits, and behavior.” ([70], p. 989). The RDoC project is a direct consequence of one of the aims of the NIMH 2008 strategic plan, namely, to “develop, for research purposes, new ways of classifying mental disorders based on dimensions of observable behavior and neurobiological measures.”[71]. Using the DSM or the ICD categories as the basis for selecting research subjects invites researchers to seek a one-to-one relationship between putative mechanisms and clinically-defined disorder categories. The goal of RDoC is to instead shift researchers towards a focus on dysregulated neurobiological systems as the organizing principle for selecting study populations. The initial stage of the RDoC project is to specify those basic dimensions of psychological functioning and their implementing brain circuits that have been the focus of neuroscience research over the past several decades. Since the ultimate goal of the RDoC project is to link dysfunctions in neurocircuitry with clinically relevant psychiatric conditions, a priority in the selection of domains is that they can be related to problem behaviors that can be found in the symptom lists of conventional disorder categories [72,73]. The preliminary RDoC working draft has identified five major domains of functioning, each containing multiple, more specific constructs: the **Negative Valence Systems** domain which includes constructs for fear, distress, and aggression, the **Positive Valence Systems** domain which includes reward seeking and learning and habit formation constructs, the **Cognitive Systems** domain which includes constructs for attention, perception, working memory/executive function, long term memory and cognitive control, the **Systems for Social Processes** domain including separation fear, facial expression regulation, behavioral inhibition, and emotional regulation constructs, and the **Arousal/Regulatory Systems** domain which include systems involved in sleep and wakefulness.

It is important to understand that the RDoC project is not intended to function as a diagnostic classification system in the way that the DSM and ICD do. Unlike the DSM, ICD, and other medical classifications which are designed to exhaustively describe and delineate the different ways that psychiatric patients might present symptomatically in terms of conceptually high-level concepts such as disease or disorder, the RDoC project is primarily a research framework to assist researchers in relating the fundamental domains of behavioral functioning to their underlying neurobiological components. As such, for each of the constructs noted above, the current state-of-the-art measurements/elements at several different units of analysis will be listed, including genes, molecules, cells, circuits, behavior and self –report [73]. Thus, in concrete terms, the RDoC framework is being implemented as a matrix, with the constructs forming the rows and the various units of analysis forming the columns. In order to fill in the various cells in the matrix, NIMH is in the process of convening a series of conferences involving experts from each of the domain areas for the purpose of refining the list of domains and constructs; this includes providing working definitions, as well as compiling for each unit of analysis a listing of the measures and components that contemporary research has identified as pertaining to a particular construct.

The RDoC approach represents a true paradigm shift in the classification of mental disorders, moving away from defining disorders based on descriptive phenomenology and instead focusing on disruptions in neural circuitry as the fundamental classificatory principle. Whether RDoC ultimately bears fruit in terms of eventually improving clinicians’ ability to predict prognosis or treatment response will depend on how well this new approach performs for research [43], something that will takes years or even decades to fully realize. But every long journey begins with a first step; taking a fresh start in the way the RDoC project is planning to do is clearly the way to go.

### Commentary

Ronald Pies, M.D.

SUNY Upstate Medical University Department of Psychiatry

Even for those of us with plentiful ego supplies, outlining an alternative to the DSM system in fewer than a thousand words poses quite a challenge! Accordingly, I would refer the reader to my more detailed comments in Bulletin 2. In that essay, I proposed the following: 1. Changing the name of our classification scheme to the *Manual of Brain-Mediated Disease*, or MBMD; 2. Conceptualizing MBMD conditions as “instantiations of disease” in so far as they entail substantial *suffering and incapacity* (dis-ease) – not as reified entities in a physical sense; 3. Separating *clinical descriptions* of disease (“prototypes”) from *research-oriented criteria;* and 4. Drawing upon six foundational principles (“The 6 Ps”), as follows:

"“Privilege” refers to strict limitations on what sort of conditions are permitted into the diagnostic schema: i.e., only conditions that entail substantial intrinsic suffering and incapacity would be “admitted” into the MBMD."

"“Prototypes” refers to the use of idealized models or archetypes of disease, rather than either “categorical” or “dimensional” methods of classification."

"“Pragmatism” refers to the instrumental nature of the diagnostic schema; specifically, psychiatric diagnosis is seen fundamentally as a means toward the effective relief of certain kinds of human suffering and incapacity."

"“Parsimony” refers to the goal usually expressed in terms of Occam’s Razor; i.e., “entities should not be multiplied beyond what is necessary.""

"“Pluralism” refers to the use of multiple types of evidence and levels of understanding in deciding what ought to count as instantiations of brain-mediated (“psychiatric”) disease."

Finally, “Phenomenology” –i.e., the *contents and structure of the patient’s felt experience*—would be an important part of the prototypical descriptions in the MBMD.

Now, I am keenly aware that the designation “brain-mediated disease” creates philosophical problems for many scholars, and this term is not to be confused with the designation *brain disease*. (Disease, as I have argued elsewhere, is properly predicated of *persons*, not of tissues or organs. See [35,74,75]). My colleague, Michael A. Schwartz MD, has suggested the alternative term “psychiatric disorders” (personal communication, Jan. 17, 2011), and others might prefer the title, “Manual of Neuropsychiatric Disease.” In my view, either term would be a vast improvement over the troublesome Cartesian vestige, “mental disorders.”

How might my proposal work for a particular condition, such as schizophrenia? Here is a greatly truncated representation of the MBMD *prototype* for schizophrenia:

"Sal is a 30-year-old single male whose chief complaint is “I can’t find pieces of me and the pieces I do have are fading, fading, fading, inter-dimensionally.” Sal’s problems began when he was about 14. According to his parents, Sal began to withdraw from friends and schoolmates and “seemed to enter a world of his own.” He became increasingly unable to maintain his hygiene, school performance, or social relations, often spending days at a time secluded in his room and refusing to shower. He would eat only foods that had been “de-contaminated from radiation,” which he believed was being “beamed” into the house. By age 18, Sal complained of “voices eating away at my brain,” and described hearing several persons discussing him in derogatory terms while alone in his room. At times, Sal would laugh or giggle inappropriately, as when attending the funeral of a family member…"

Of course, the “real” MBMD would provide a much more detailed and comprehensive prototype. When warranted by epidemiological and clinical data, prototypical descriptions of disease *subtypes* would be provided; e.g., “paranoid” or “catatonic” subtypes of schizophrenia (though arguably, these subtypes are not adequately supported by existing studies). In addition, under the rubric of “Ancillary Findings,” the MBMD would provide data on prototypical neuropsychological findings; brain imaging and related neurophysiological data; and a rich description of the patient’s “inner world,” such as provided by Silvano Arieti in his classic text, *Interpretation of Schizophrenia*[76]. The general nature of the “suffering and incapacity” would be described in detail for a given condition. There would be no specific “necessary and sufficient” criteria for applying a diagnosis, in the manner of the “3 from column A, 2 from column B” approach of the DSMs; rather, the clinician would be prompted to decide if the overall description of the prototype and its ancillary findings generally fit well with the patient’s history, mental status exam, and experience of the world. A list of exclusion criteria would also be provided; e.g., “the patient’s condition is not more likely due to brain injury, intoxication, substance abuse,” etc., though these could well be co-morbid diagnoses. In short, the prototypes would be “fuzzier” than the *research diagnostic criteria* provided in a separate part of the MBMD, but would be *consistent* with those criteria. (Readers old enough to remember the DSM-II (1968) will probably be amused at the superficial resemblance of my MBMD prototypes to descriptions in the DSM-II – ironic, but quite intentional! However, unlike the DSM-II descriptions, the MBMD prototypes would be written from a *patient-based* perspective).

Finally, I would like to see the MBMD simplify the overall psychiatric disease classification. I believe that the vast majority of clinically-significant instantiations of brain-mediated disease could fit into one of the following general categories:

Disturbances of Mood, Affect and Emotion

Disturbances of Reality Perception and Psychic Integration

Disturbances of Attention, Cognition and Memory

Disturbances of Appetitive Behavior or Impulse Control

Disturbances of Interpersonal Relations and Social Adaptation

Disturbances with “mixed” or overlapping features

Ultimately, I would envision an integration of these general categories with emerging data on endophenotypes, biological markers, and brain neurocircuits. But this should not diminish the emphasis the MBMD will give to phenomenology: the unique “felt experience” of the patient.

I use the term “brain-mediated” in two senses. The “loose”, ordinary-language sense holds that a condition or disease is brain-mediated if most salient aspects of the condition are explained by, or associated with, the function and dysfunction of the brain, and not some other organ. On this view, both schizophrenia and epilepsy are instantiations of “brain-mediated disease.” Alternatively, the “strong form” of the argument holds that a condition is “brain-mediated” when the brain is both *necessary and sufficient* to account for the relevant “inputs” and “outputs” of the condition. “Inputs” refer to the proximate etiological factors (e.g., abnormal dopamine levels, head trauma, etc.) leading to disease; “outputs” refer to the experiential and behavioral expressions of the condition (e.g., hallucinations, delusions, seizures, etc.). The “strong” definition – for reasons beyond the scope of this piece – is clearly harder to defend than the ordinary-language version.

### Acknowledgement

Thanks to Michael A. Schwartz MD for his reading of an earlier version of this piece; and to Robert Daly MD, for his stimulating essays on the nature of “madness”.

**Further readings**: [35,74,75,77-82]

Commentary

Joel Paris, M.D.

McGill University Department of Psychiatry

The introduction of widespread dimensional measures into DSM-5 involves this as well as the previous question. Revisions of the DSM system must give a strong priority to clinical utility. When diagnoses become too complex, they will not be made, and patients suffer the consequences. The more complicated features proposed for DSM-5, such as dimensional scales, should be reserved for research purposes. Clinical diagnosis, which is carried out by busy practitioners, benefits from streamlining and simplicity.

Specifically, the dimensionalization of diagnosis proposed for personality disorders, as well as the proposals for scales to measure functioning, and for scales that assess disorders within a wider pathological spectrum, all lack clinical utility. Clinicians will not be interested in dimensions unless they are measured in a practical way, as for example, blood pressure. Asking them to score patients on multiple dimensional scales is impractical, and as First points out [39], the consequences of failure of usage due to poor utility could be even more serious than leaving the system unrevised.

This problem raises another question: why does the DSM system not consider separating the use of its manual for clinical and research purposes. Clinicians do not have time to make diagnoses using complex procedures, and should not be expected to spend more than a few minutes making a decision. Researchers, on the other hand, have the luxury of using complex measure to be precise, and need to have measures that have strong scientific validation. Failure to separate these uses of the manual is a deficit that affects the entire system.

### Allen Frances responds: Alternate universes must prove themselves

Every month or so, someone (usually very smart and passionate) sends me a detailed proposal for a new diagnostic system offered as an alternative to the jumbled, pedestrian, atheoretical, and purely descriptive method used in DSM. The new system is invariably theory driven, clever, neat, and plausible. Surely, it is quite easy to be more coherent than a DSM that consists of a jumble of disorders gathered together largely through a historical accreting process based mostly on clinical observation and descriptive research – without an underlying theory or deep knowledge of causality.

The new systems come in 3 types: 1) Brain biology – these used to be based on correlates with neurotransmitters, but recently neural networks of various kinds are much more popular; 2) Psychological dimensions – hundreds of scales have been developed and carefully tested; and 3) Systems based on psychodynamic, ethological, and developmental models – less popular now than they once were.

Unfortunately, none of these approaches, however elegant, is remotely ready for inclusion in the official system of psychiatric nomenclature. DSM must by its very nature be a conservative document that follows and never leads the field. The problem with all of the suggestions to replace the admitted DSM jumble is that there are so many contenders, none of which has been proven or has attained wide acceptance from the field. It is also not possible to choose one from among so many plausible, but necessarily parochial systems, when most clinicians have absolutely no interest in any of them and the proponents of rival systems can make about equally valid claims for their respective pet methods.

The DSM-IV experience with the personality disorders was a rude and disheartening awakening. I very much hoped to include a (at least optional) dimensional personality rating scale. We were able to gather together in one room the proponents of all the competing dimensional systems to attempt the selection of one or some compromise among them. It didn't work – we could not forge a consensus because each participant remained wedded to his own scale (however minimally different it was from its near neighbors). Without wide agreement, it is impossible to force a field to accept changes that represent one necessarily narrowly defined perspective. The DSM-5 effort to include personality dimensions will also undoubtedly fail – for this reason as well as for its unbelievably byzantine complexity.

I feel sure that our clumsy descriptive classification may not be the only, or even the optimal way, to sort things for future research. But I feel equally certain that the DSM remains necessary to carry forth the current, everyday, practical clinical and administrative work that are its first priority. Once we have attained a widely accepted, etiological understanding of at least some forms of psychopathology, the new insights will gradually replace our clumsy, but nonetheless now still useful system.

At this stage in this arena, the wisdom of the philosopher Vico trumps the much greater and better known Descartes. Descartes sought to use what we now call Cartesian rationality and mathematical order to sort what were previously seemingly disorderly phenomena. This turned out to be a screaming success in the mathematical, physical and chemical worlds, but has (as Vico predicted) much less purchase in understanding the sloppy complicatedness of human affairs – including psychiatric diagnosis.

**Response to Dr First**: I agree that NIMH's RDoC project is the best hope for revolutionary advances in psychiatric diagnosis and in our understanding of psychopathology. But the obstacles are huge. The complexity of the brain has dwarfed the reach of even our most powerful research tools. Our science will advance, but probably will uncover vast new territories of our ignorance for every new beachhead of new knowledge. It may take decades of concerted effort for this project to bear clinical fruit and impact on the diagnostic system. It is an open question whether NIMH will be able to mount the necessary sustained commitment. Many things could derail the future momentum of this project – budget constraints, a new director with different goals, a lack of cooperation among scientists, a lack of findings or findings that caste things in a different light. RDoC is indeed our most promising seed – let us hope it grows and thrives. But the prospects for its future success are unpredictable in these early days.

**Response to Dr Pies**: Dr Pies offers a plausible possible alternative to DSM diagnosis, but certainly not one that is compelling enough to cry out for acceptance. There are obvious problems: 1) Dr Pies' prototypal system of clinical diagnosis is a throwback to DSM II and would likely create the familiar problems of low reliability and a complete disconnect between clinical practice and research; 2) We agree that DSM may be too much a splitter's system, but Dr Pies' big lumps would fail to capture the complexity and heterogeneity of clinical presentations; and, most important; 3) there are no compelling data to support that the system would work. In the absence of scientific advance supporting and requiring change, this exercise in reorganizing our tired descriptive psychiatry feels like furniture rearrangement – not likely to further our field.

**Response to Dr Paris**: I agree completely re the foolishness of the DSM-5 dimensions, but place more value on having one system used by both clinicians and researchers.

## Conclusion

As with the previous four questions, commentaries and responses on the final two offer a mix of opinion, with both agreement and occasional disagreement. Regarding the fifth question, utility, Frances agrees with the specific critique of the proposed DSM-5 dimensional measures as offending clinical utility, but not with the more general critique of DSM-III & IV as embodying unresolved conflicts between clinical and research utility. He is, after all, responsible for the DSM-IV statement:

The utility and credibility of *DSM-IV* require that it focus on its clinical, research, and educational purposes and be supported by an extensive empirical foundation. Our highest priority has been to provide a helpful guide to clinical practice. We hoped to make *DSM-IV* practical and useful for clinicians by striving for brevity of criteria sets, clarity of language, and explicit statements of the constructs embodied in the diagnostic criteria. An additional goal was to facilitate research and improve communication among clinicians and researchers. ([33], p. xv).

Let’s first recognize that the fifth question, “Is there a conflict over utility in the DSMs?,” contains in fact three questions: is there a conflict among the various goals?, what goal or purpose is served best?, and would we have been better off with more than one manual? Frances tends to acknowledge the first, ignore the second, and center his argument around responding to the third question in favor of a single manual. He concludes that “[h]owever imperfect, DSM's special value is as a common denominator that avoids a Babel and is good enough (if admittedly not best) at each of its jobs.” Unlike the pluralogue in the other questions, where some or most of the commentators were on Frances’ side of the argument, in the question of utility we have all three commentators lined up against him: all recognizing the clinical/research conflict, all agreeing that DSM-IV is prejudiced toward research, and two of the three opting for separating clinical and research diagnostic documents.

With their agreement that there is a conflict between the clinical and research goals of the DSM, the three commentators offer different perspectives on this conflict. Adopting Aristotle’s distinction between theoretical and practical knowledge (*episteme* and *phronesis*), Owen Whooley argues that there is a rather deep, metaphysical divide between the research and clinical goals of DSM-III/IV: the search for universal laws versus individualized care of the particular patient. He illustrates this vividly with the proposed dimensional measures for DSM-5. Aside from the practical matters such as that clinicians will find them cumbersome, of no practical use, and won’t use them, they also represent, philosophically, an effort to treat human beings and human suffering as quantifiable entities who can be evaluated with quantitative measures as opposed to interpretation and judgment.

Joseph Pierre focuses on what he might call the DSM architects’ own confusion regarding the goal and use of the manual. While they subject the clinician to the diagnostic criteria in the service of promoting research, the categories with their criteria impede research as much as they facilitate it. Citing the RDoC, Pierre points out that the most significant research may be done outside the confines of the DSM categories. Finally, Aaron Mishara and Michael Schwartz point to the clinical/research conflict as a consequence of basing DSM-III on a Hempelian scientific model; they argue that a DSM designed with the ideal-type structure they advocate would eliminate the clinician/researcher split and would in fact serve the two groups equally well.

Finally this six-question exercise ends appropriately with a grand question (related to the earlier question regarding attitudes toward change): do the problems of the DSMs warrant a major overhaul? Consistent with his previous response arguing for a conservative attitude toward change, Frances argues that the state of psychiatric science dictates minimal change, not the “paradigm shift” proposed by the DSM-5 architects, and not any other form of major overhaul. He invokes the NIMH Research Domain Criteria project (RDoC) that promises to change the scientific landscape of psychiatry in the future. Pending findings from that endeavor, which may indeed warrant a significant refashioning of the DSM, we should hold tight and await the return of the RDoC jury.

The first commentary flows neatly from Frances’ closing remark. Michael First provides a clear description of the NIMH project, clarifying that this is research project, not an alternative diagnostic manual. But it is a research project whose findings may significantly affect all DSMs that follow in its wake. In a second commentary Ronald Pies reviews his effort at imagining an alternative diagnostic system, described more thoroughly in Bulletin 2. His proposal involves two innovations: basing the system on prototypal diagnostic constructs, and dramatically reducing the number of diagnoses from several hundred to a large handful. Finally, Joel Paris tackles the major innovation of DSM-5, the introduction of a variety of dimensional measures. His critique overlaps with some of the discussion in the previous question on utility – for the obvious reason that the introduction of such measures involves both questions, utility and alternate systems. He is in agreement with previous discussion of this topic – indeed, we have not had a positive response, either from Frances or any of the commentators, toward the proposed dimensional measures. Paris also expresses an agreement with commentators of Question 5, that the manual might work better by being split into two: a shorter version for clinicians and a more detailed version for researchers. Such a split would certainly work for Pies’ proposed alternative system, and it again touches on the issue of utility.

It is around question 5, utility, that Allen Frances and I have been in most disagreement. He certainly recognizes that DSM-IV will be dissatisfying to each of its users in its own way; but committed as he is to a single manual, he is probably right that DSM-IV does a reasonable job of reconciling the diverse goals of its different interest groups. I do remain convinced that for their work clinicians only need prototypal descriptions (which are in fact included in DSM-III/IV), although I can also agree that the criteria have played a role in reinforcing the prototypes. For me the diagnostic criteria have had a complicated course. They certainly play a role in reinforcing reliability across clinical and research settings. That accomplished and the prototypes in place, they lose their usefulness for clinicians. With regard to the research community, the diagnostic criteria have played an ambiguous role: on the one hand assuring reliability across research settings, on the other hand restricting research by forcing research to work within the criterial boundaries. I agree with Owen Whooley that the manual with its diagnostic criteria and its proposed dimensional measures does expose a deep discord between conflicting visions of people and psychopathology. I also agree with Joseph Pierre regarding a core contradiction in the DSM structure: that it is designed to support research but in the end imposes strictures that force researchers to work outside the manual. Finally, I am sympathetic to the way in which Aaron Mishara and Michael Schwartz’s analysis converges with Pies’ in the next section, the restructuring of the manual around a small group of superordinate diagnostic categories. This is of course the lumping strategy; I am not convinced that the DSM approach of splitting into hundreds of diagnoses has accomplished much.

With question 6 I can in some fashion agree with everyone. Allen Frances makes a convincing case that, pending more definitive science than currently available, we should stick with the DSM that we have, and, in agreement with Joel Paris, without the dimensional measures currently planned. But if there is to be a more definitive science, the NIMH Research Domain Criteria project as described by Michael First seems like the best prospect. What we all agree about – Frances, the commentators, and myself – is the uncertainty as to whether the RDoC project will succeed in becoming the real paradigm shift it promises to be.

## Competing interests

MF is an external consultant to the NIMH Research Domain Criteria (RDoC) Project. NG has research grants from Pfizer and Sunovion, and is a research consultant for Sunovion. MS is a consultant for AstraZeneca, Merck, and Sunovion. Other authors report no competing interests.

## Authors’ contributions

JP (Phillips) wrote the general General Introduction and Conclusion, as well as the introductions to the individual conclusions. AF wrote the Responses to Commentaries. MC, JC, HD, MF, NG, GG, AH, WK, SL, EM, AM, JP (Paris), RP, HP, DP, CP, MS, TS, JW, SW, OW, PZ wrote the commentaries. All authors read and approved the final manuscript.
